# Sleep promoting and omics exploration on probiotics fermented *Gastrodia elata* Blume

**DOI:** 10.1038/s41538-024-00277-8

**Published:** 2024-06-18

**Authors:** Chao-Qi Zhang, Xu-Dong Zhang, Yan Wang, Yi-Han Liu, Cun-Li Zhang, Qiang Zhang

**Affiliations:** 1 Shaanxi Key Laboratory of Natural Products & Chemical Biology, College of Chemistry & Pharmacy, Northwest A&F University, Yangling, 712100 China; 2 Key Laboratory of Edible Plant Enzyme R&D and Monitoring, Shaanxi Wuding Biotechnology Co., Ltd., Hanzhong, 724400 China

**Keywords:** Metabolomics, Nutrition

## Abstract

Fermenting Chinese medicinal herbs could enhance their bioactivities. We hypothesized probiotic-fermented *gastrodia elata* Blume (GE) with better potential to alleviate insomnia than that of unfermented, thus the changes in chemical composition and the insomnia-alleviating effects and mechanisms of fermented GE on pentylenetetrazole (PTZ)-induced insomnia zebrafish were explored via high-performance liquid chromatography (HPLC) and mass spectroscopy-coupled HPLC (HPLC-MS), phenotypic, transcriptomic, and metabolomics analysis. The results demonstrated that probiotic fermented GE performed better than unfermented GE in increasing the content of chemical composition, reducing the displacement, average speed, and number of apoptotic cells in zebrafish with insomnia. Metabolomic investigation showed that the anti-insomnia effect was related to regulating the pathways of actin cytoskeleton and neuroactive ligand-receptor interactions. Transcriptomic and reverse transcription qPCR (RT-qPCR) analysis revealed that secondary fermentation liquid (SFL) significantly modulated the expression levels of neurod1, msh2, msh3, recql4, ercc5, rad5lc, and rev3l, which are mainly involved in neuron differentiation and DNA repair. Collectively, as a functional food, fermented GE possessed potential for insomnia alleviation.

## Introduction

Insomnia is a commonly observed sleep disorder, predominantly defined by challenges in starting sleep and experiencing suboptimal sleep quality, and often manifests as comorbidity with depressive disorders^1,2^. In the long run, insomnia will seriously affect people’s life quality and physical and mental health^3–5^. Sleep is closely linked to a variety of metabolic dysfunctions in humans. Sufficient sleep is essential for restoring the functions and metabolism of nearly all tissues and cells in the body, promoting optimal metabolic health^6,7^. Dysfunctions in the hypothalamic-pituitary-adrenal axis, central neurotransmitter systems, vagal tone, melatonin system function, inflammatory response factors, and limbic cortical system circuits have been proposed by researchers to be the main causes of insomnia^8,9^. The alteration of hypothalamic-pituitary-adrenal (HPA) axis activity is one of the most common neuroendocrine abnormalities in patients with mental disorders^10^. The synthesis and release of cerebral cortisol, the expression level of melatonin concentration, and the expression of anti-inflammatory cytokines are the main causes of insomnia^11^. Their molecular effects are processed, elaborated, and further translated into signals that may point to peripheral (i.e. autonomic and endocrine effects) as well as to the brain (i.e. effects on neurotransmitter synthesis and release)^12^. However, further research is required to better elucidate the pathogenesis of insomnia for better treatment. Sleep disorders are often managed with a range of medications, such as benzodiazepines, nonbenzodiazepine omega-receptor agonists, tricyclic antidepressants, selective GABA agents, and antihistamines. Nevertheless, these sedative medications may cause serious adverse effects^13^. Overdose deaths involving benzodiazepines increased between 1999 and 2015^14^. Nonpharmacological interventions are currently being recognized as the preferred initial approach for managing chronic insomnia^15^. Functional food or healthcare food can be served as an important approach to relieve insomnia symptoms, such as *Schisandra Chinensis* Fructus, and Polygalae Radix. GE is a botanical specimen originating from China, which holds significant historical significance in the realms of traditional medicine and culinary traditions^16^. Phenols, glycosides, adenosine, and its analogs, particularly p-hydroxybenzaldehyde, are the main sources of sedative and hypnotic effects of GE^17,18^. Interestingly, GABA_A_-Rs is the target of benzodiazepines and is known to exert hypnotic effects by enhancing its function^19^. In addition, GE may also regulate sleep by turning on adenosine A_1_/A(2 A) receptors and stimulating the VLPO sleep center^20^. It has been reported that para -hydroxybenzyl alcohol (HBA) and poly(3-hydroxybutyrate) (PHB) possess the capability to induce anxiolytic effects in the context of sedation and hypnosis by interacting with the 5-HT system and γ-GABA system, respectively^18,21^.

Chinese medicinal herbs have been used for centuries to treat diseases and are vital for human well-being. Moreover, Chinese medicinal herbs typically contain low contents of bioactive compounds^22^. Fermentation not only leverages the bioactivity of probiotics to alter the nutritional composition and bioactive compounds of plant-based foods, but also has great benefits for gut microbiome composition and the immune system^23^. However, there have been no reports on the sleep-promotion function of probiotic-fermented GE yet. Furthermore, our preliminary experimental results show that the probiotics-fermented GE showed a higher content of active ingredients than unfermented GE. In light of the above considerations, it could be reasonably hypothesized that probiotics rely-fermented GE has the potential to be a functional food with insomnia-alleviating effects.

Herein, the study aims to investigate whether probiotic-fermented GE has superior insomnia-alleviating effects compared to unfermented GE. And we applied transcriptomic and metabonomic analysis of insomnia Zebrafish treaded with fermented GE to explore the essential regulations invoked by GE.

## Results

### Neuroprotective effect against low dose PTZ-induced mental injury

**Fig. 1:**
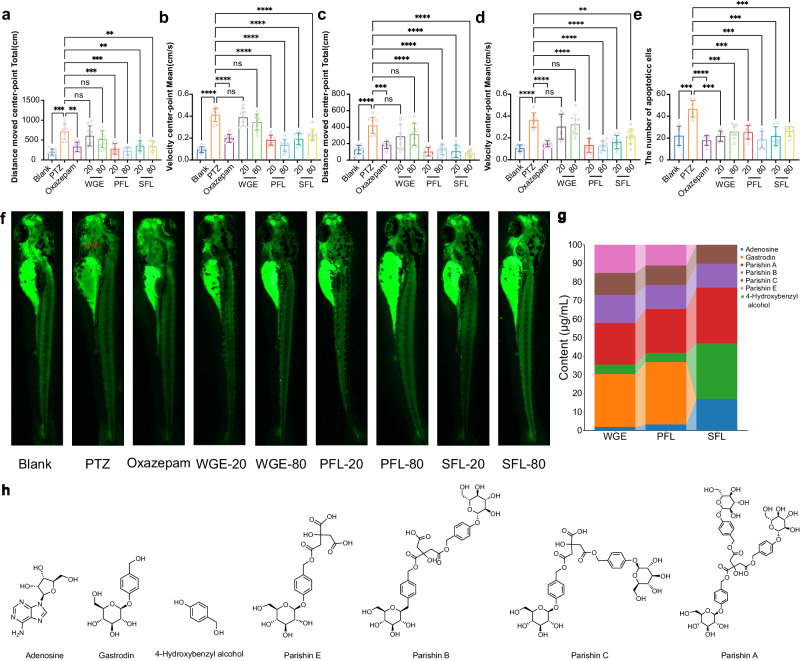
Activity evaluation and analysis of main active ingredients of fermented *Gastrodia elata* Blume. **a**–**e** Effects of GE samples treated at different stages on PTZ-induced insomnia-like zebrafish behavior. Data are presented as means ± standard deviation (s.d.) (*n* = 9); **P < 0.01, ***P < 0.001, ****P < 0.0001) and were analysed using one-way ANOVA followed by Tukey’s multiple comparisons tests. **f** Zebrafish brain cell apoptosis. **g** Dynamic changes of the main chemical constituents of GE samples during fermentation. **h** Chemical structure of the main components.

Zebrafish exhibit neural networks analogous to those found in mammals, and their locomotor activity represents a measurable and intricate behavioral phenotype^24^. To confirm the effect of GE samples at each treatment stage on animal behavior and nervous system damage, we measured the total travel distance and swimming speed of zebrafish for 30 and 50 min, and the amount of apoptotic cells. As shown in Fig. 1a–d, the total distance traveled and mean speed were significantly increased in PTZ-exposed larvae. The behavioral changes of zebrafish larvae were significantly reversed after pretreatment with GE samples at different processing stages. Nevertheless, there were no statistical differences between the low-dose and high-dose GE samples. In general, prime fermentation liquid (PFL) and SFL samples had similar effects on zebrafish movement behavior, including total movement distance and average movement speed. Additionally, PFL and SFL samples were significantly more effective than WGE samples in improving the movement behavior of zebrafish with insomnia. Obviously, fermentation process enhanced the sedative and sleeping biofunctions of GE.

Sleep deprivation has a significant influence on the nervous system. Lack of sleep can cause an imbalance in DNA damage and repair^25^. Anti-apoptotic activity is an important cause for the neuroprotective effects of sleep-related disease drugs^26^. Apoptosis in the nervous system is an important reflection of nerve damage. The apoptotic cells in zebrafish larvae can be visualized through fluorescent staining using a fluorescence microscope, owing to the transparent nature of their bodies. Apoptotic cells within the nervous system had a green fluorescence following staining with Acridine orange (AO). Figure 1f illustrates a significant presence of apoptotic cells in zebrafish brain of the PTZ group. Different processing stages of the GE sample considerably decreased the number of apoptotic cells observed in the brain of zebrafish. GE sample effectively inhibited PTZ-induced apoptosis in the larvae brains. Thus, fermented GE provided more nerve protection than unfermented GE.

### Main components change in GE fermentation

To investigate the dynamic change of chemical compounds, we comparatively analyzed the GE samples of water extract of GE (WGE), PFL, and SFL. The results indicate that the content of four active ingredients (parishin B, C, E, and 4-hydroxybenzyl alcohol) remained stable from stage WGE to stage PFL. The contents of five medicinal active ingredients (adenosine, 4-hydroxybenzyl alcohol, parishin A, B and C) significantly increased from stage PFL to stage SFL (Fig. 1g). In particular, the concentration of 4-hydroxybenzyl alcohol, parishin B, C, and E in the SFL stage increased 19.7-, 2.9- 2.9-, and 2.9-fold, respectively, compared to stage WGE. However, it is worth noting that parishin A and gastrodin showed a brief decline from WGE to PFL, and decreased to 0 from PFL to SFL. Adenosine increased briefly from WGE to PFL stage and then rapidly increased in SFL stage (245.72 ± 12.53 μg/mL), increased 28.9-fold, compared to stage WGE (Fig. 1g and Supplementary Table 2). During the fermentation process, the contents of seven active ingredients underwent significant changes. Among them, parishin E and gastrodin were not detected in SFL sample. The remaining five active components demonstrated a notable overall increase. These content variations indicated that probiotics fermentation invoked active components GE changing. Similar exploration has also found that probiotics can influence changes in active ingredients^27,28^. Therefore, probiotic fermentation could be a useful processing method to enhance the biological functions of ingredients or herbs. However, this enhancement may vary according to ingredient material and probiotic strains and more exploration is still needed.

### LC-MS/MS Profiling of GE samples at different fermentation stages

**Fig. 2:**
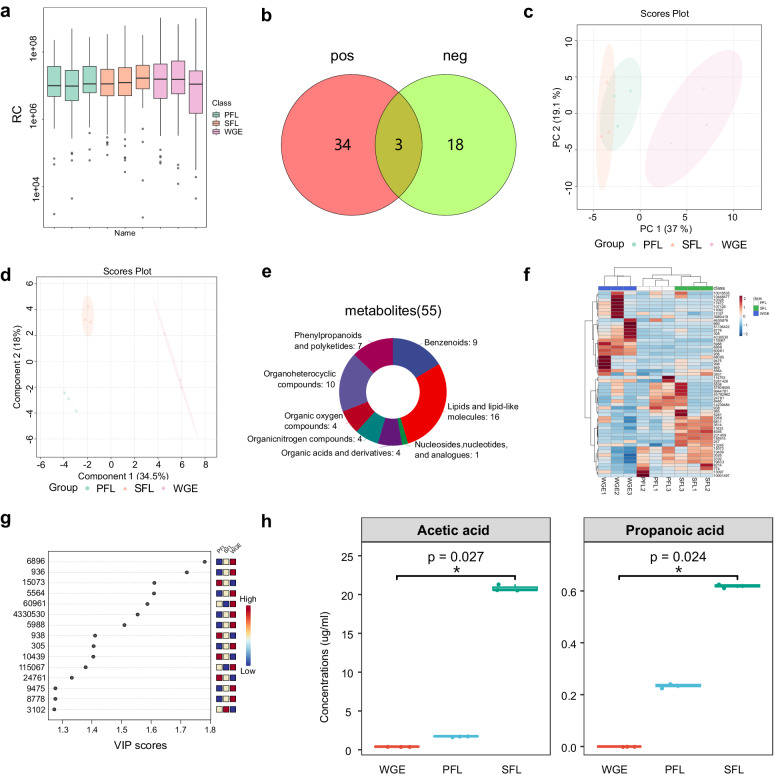
LC-MS/MS analysis of GE samples at different treatment stages. **a** The box plot of Component in WGE, PFL, and SFL groups. **b** The quantity of chemicals found in both the negative (neg) and positive (pos) ion modes of the ESI. **c**, **d** PCA and sPLS-DA examination of samples at different treatment stages. **e** Types and proportions of the compounds identified from WGE, PFL, and SFL. **f** Heatmap visualization. **g** VIP value obtained from the OPLS-DA model of the potential markers in WGE, PFL, and SFL. **h** The changes of SCFAs in samples at different treatment stages; Data are presented as means ± standard deviation (s.d.) (*n* = 3); *P < 0.05) and were analysed using one-way ANOVA followed by Tukey’s multiple comparisons tests.

To profile chemical component changes in fermented GE, we used LC-MS/MS analysis. Figure 2a presents the aggregation of metabolite overall expressions for each sample. A total of 55 chemical components were detected from positive and negative ions using ESI. ESI positive and negative resources yielded the identification of only three metabolites (Fig. 2b). The results disclosed obvious variations among GE samples at different fermentation stages, as shown in Fig. 2c, d. PCA analysis displayed 71% features across samples and each group had strong clustering tendencies and maintained distinct separation from one another.

In the present study, these metabolites were identified in the samples at three distinct processing stages that encompassed nine benzenoids, 16 lipids and lipid-like molecules, one nucleoside, nucleotide, and analog, four organic acids and derivatives, four organic nitrogen compounds, four organic oxygen compounds, 10 organoheterocyclic compounds, seven phenylpropanoids, and polyketides. This information is visually represented in Fig. 2e. Lipids and lipid-like compounds made up the largest group among them, making up 29.09% of the total metabolite composition in terms of relative concentration.

To further understand the impact of fermentation process on the active components in GE, we performed PLS-DA analysis. Hierarchical cluster analysis (HCA) showed how metabolites accumulated in fermentation processing in Fig. 2e. The Euclidean distance arithmetic was used to cluster the 55 discovered metabolites of GE samples in heat maps, as illustrated in Fig. 2f. The dendrogram classified the metabolites found at various heat processing stages into three groupings. The higher concentration of a specific metabolite in the corresponding sample is indicated by the brighter hue. The HCA heat map revealed more variations in abundance between the PFL and WGE samples than between the PFL and SFL samples, suggesting that the metabolites in the fermented liquid may undergo distinct changes throughout the fermentation phase, and the abundance of PFL and SFL samples is similar, probably because of their fermentation time. Nevertheless, in general, the contents of chemical components before and after fermentation changed dynamically. Compared with WGE, SFL and PFL significantly increased the contents of 24 and 19 compounds respectively. Among them, 12 compounds were detected in both (Supplementary Fig. 1a, b). Benzenoids, lipids and lipid-like molecules, organic nitrogen compounds, and organoheterocyclic compounds are the major super classes. This may be one of the main reasons why the PFL and SFL have similar phenotypic results. In addition, SFL also shows unique fermentation advantages compared with PFL. These compounds classes that SFL improved include organic acids and derivatives, benzenoids, lipids and lipid-like molecules, organoheterocyclic compounds, phenylpropanoids, and polyketides. the direct parent compounds are alpha amino acids, benzamides, medium-chain fatty acids, phthalic anhydrides, coumarins and derivatives, cinnamic acids, and stilbenes (Supplementary Table 3). The VIP score chart shows that the top five compounds are 2,6-dimethylaniline, nicotinamide, 3-methylpyrazole, triclosan, and adenosine. However, the top four compounds have nothing to do with the sleep-promoting activity. The fifth-ranked adenosine, one of the primary active components of GE (Fig. 2g), has a large contribution rate in different processed GE samples, and the research has shown that the adenosine analog could be a potential hypnotic^29^. From the GE processing efficiency perspective, adenosine may become a new entry point for GE research. Short-chain fatty acids can participate in many physiological functions and are thought to affect sleep through the gut-brain axis pathway^30^. Herein, we investigated the impacts of fermentation on short-chain fatty acid (SCFA) accumulation, as shown in Fig. 2h. we detected only acetic acid and propionic acid in the samples at different fermentation stages. It is worth noting that the content of acetic acid and propionic acid also increased sequentially as the fermentation progressed. It could be seen that changes in chemical composition content were related to fermentation technology, which may be one of the reasons for the change in the phenotypic results.

### Metabolomic profiling of insomnia larvae exposed SFL

**Fig. 3:**
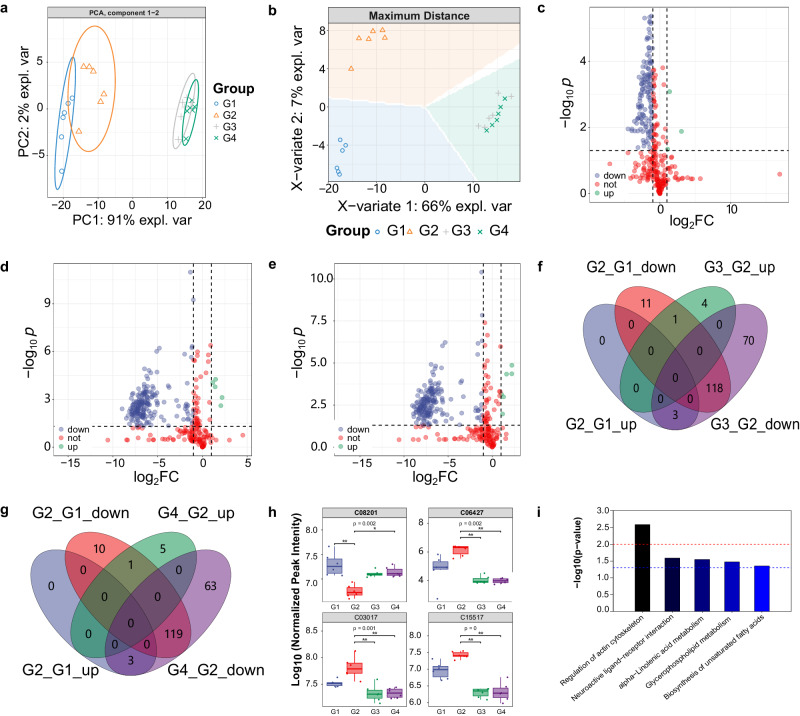
Metabolome analysis of zebrafish. **a** PCA and (**b**) sPLS-DA analysis of LC-MS profiles, *n* = 6 per group. **c**–**e** Differences in metabolites between G1 and G2, G2 and G3, and G2 and G4 are represented by volcano graphs. Every point in the volcano plots is a metabolite. **f**, **g** The number of significantly changed differential metabolites among the three groups of G1, G2, G3 and G4. **h** The relative amounts of the important differential metabolites; Statistical significance: Kruskal–Wallis test followed by Dunn’s post-hoc pairwise comparison, *p < 0.05, **p < 0.01; (*n* = 6). **i** Pathway enrichment.

Insomnia is closely related to changes in metabolic function^31^. To investigate the metabolomic influence of fermented GE, we then carried out LC-MS/MS profiling on metabolome variation on SFL-treated, insomnia, and blank control groups. As shown in Supplementary Fig. 2. In the positive ion mode, 203 metabolites were detected, whereas in the negative ion mode, 204 metabolites were discovered. Among them, 66 metabolites were identified in both modes. PC1 and PC2 were able to capture 93% of the sample features, as indicated in Fig. 3a. Furthermore, a considerable change in metabolites across treatments was indicated by the relatively high gap between groups. Each group was predicted by the sPLS-DA analysis in Fig. 3b. As a result, there were significant differences in the metabolic data between the groups when using different treatments. The groups treated with 20 µg/mL SFL(G3) and 80 µg/mL (G4) showed considerable overlap in PCA and PLS-DA analysis plots. This suggests that the metabolic levels of the two groups were very close to each other.

We then conducted pairwise comparisons to collecte metabolites of upregulation or downregulation between G2/ G1, G3/G2 as well as G4/G2, as shown in Fig. 3c–e. The altered metabolites were enumerated and visually shown in the venn diagram (Fig. 3f, g). Four important differential metabolite expression levels were redressed by both low and high-dose SFL, which were the hyodeoxycholate (C00319), propanoylcarnitine (C03017), alpha-linolenic acid (C06427), acetylcholine chloride (C08201). The G2 raised the levels of three metabolites and lowered the levels of one, as shown in Fig. 3h, yet these four differential metabolites changes were significantly reversed in both G3 and G4 groups.

To gain a better understanding of how SFL exposure induces metabolite changes, we performed metabolic pathway enrichment analysis to explore and visualize metabolic pathways affected by SFL. The enriched pathways observed in Fig. 3i encompass the regulation of actin cytoskeleton (dre04810), neuroactive ligand-receptor interaction (dre04080), glycerophospholipid metabolism (dre00564), alpha-linolenic acid metabolism (dre00592), biosynthesis of unsaturated fatty acids (dre01040). The *p*-value associated with the metabolic path indicates the level of statistical significance on the enrichment of the pathway. The top one significantly enriched pathway is the regulation of actin cytoskeleton (dre04810). Traditional Chinese medicine can improve insomnia by the regulation of actin cytoskeleton, and the aforementioned pathway assumes a critical function in neuroprotection and regulating cell proliferation and differentiation^32,33^.

### Transcriptomic analysis on regulations of SFL

Transcriptomic profiling can provide a system view on mRNA variation influenced by SFL. To evaluate if a given set of genes exhibits statistically significant, consistent difference between two biological states, a computer method was applied. A statistical technique called gene enrichment analysis, also known as functional enrichment analysis is used to find genes that are overrepresented in a large gene set and may be connected to particular disease characteristics^34^.

We used GSEA to investigate the impact of SFL on gene function. Gene ontology (GO) biological process function annotation was combined to screen the gaps between 15 and 500 genes in the GO entries. Then, the absolute value of the normalized enrichment score (NES) > 1, FDR *q*-value < 0.25, and the nominal *p* value < 0.05 were deemed as the significantly enriched pathways. These gene sets were ranked from highest to lowest in absolute value of NES, and the top gene sets were randomly selected. Positive and negative NES indicated higher and lower expression in G4 or G2, respectively.

**Fig. 4:**
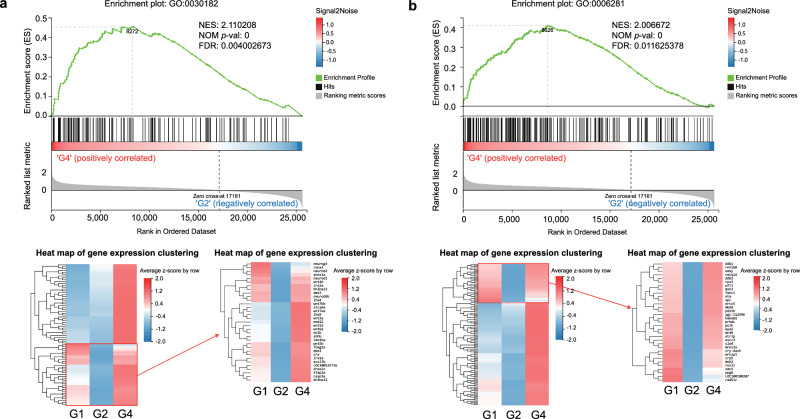
GSEA was performed in the G4 and G2 groups and G2 and G1 groups. In a ranked list of all the genes in the RNA-seq dataset, the GSEA algorithm generates an enrichment score that indicates the extent of overrepresentation at the top or bottom of the list of genes included in a gene set. Positive enrichment scores (ES) indicate gene set enrichment at the top of the ranked list, whereas negative ES indicate gene set enrichment at the bottom of the ranked list. Heatmaps beneath each enrichment plot show the expression heatmap signatures of genes involved in the GSEA analysis, referring to enrichment plots (**a**), (**b**). Left: the clustering of all genes within the pathway. Most sensitive pathways are highlighted at the right (indicated by a red square in panel 4). The red color is used to represent high expression, whereas the blue color is used to represent low expression. The analysis demonstrates that known (**a**) neuron differentiation, and (**b**) DNA repair, are enriched in G4 groups.

We found that the peripheral nervous system development, neuron differentiation, and DNA repair were more highly expressed in the G4 Group (Fig. 4a, b and Supplementary Fig. 3a). However, the ‘Negative regulation of mitochondrial electron transport, cytochrome c to oxygen’ and the ‘negative regulation of organic acid metabolic process’ were more highly expressed in G2 Group (Supplementary Fig. 3b, c). The ‘neutrophil chemotaxis’ and ‘regulation of neurogenesis’ were more highly expressed in G2 Group (Supplementary Figs. 3d, 4a). On the contrary, the ‘negative regulation of visual perception’ was more highly expressed in the G1 Group (Supplementary Fig. 4b). The above results indicated that the related pathways should deserve more attention in further research. Next, 133 genes with significant changes among the groups were screened out on the above-related pathways (Supplementary Table 5); however, which gene(s) among the 133 genes plays a key role is unclear. Thus, we carry out key driver gene analysis (KDA) to help understand the genes in the main regulatory position among the selected genes.

### KDA analysis

**Fig. 5:**
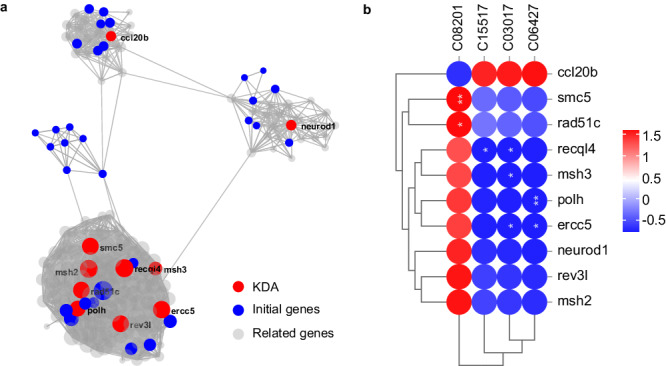
Key driver gene analysis. **a** Key driver analysis of all genes differentially expressed in pathways. The genes labeled red are the key driver genes. Parameter interpretation: key gene (KDA) refers to the gene with a key role in the gene set. The key gene may come from the selected gene or from a gene with a PPI relationship with the selected gene under the corresponding PPI score parameter. Initial gene: The non-critical gene (KDA) of the initially selected gene. Related genes: Genes with a PPI relationship with the selected gene (excluding key genes) under the corresponding PPI score parameter. **b** Correlation coefficient clustering heat map. The abscissa represents metabolites, the ordinate represents genes, * represents a *p* value of < 0.05 in the correlation coefficient significance test, and ** represents a *p* value < 0.01. The color labels represent Pearson correlation coefficient values.

A total of 133 genes were selected for investigation, we predetermined the number of key genes and linked genes, as well as set the protein-protein interaction (PPI) score. Subsequently, a KDA analysis was conducted. As shown in Fig. 5a, interestingly, we found ten key genes through KDA; among them, ten key driver genes such as neurod1, rev3l, msh2, recql4, ercc5, msh3, polh, smc5, and rad51c, are involved in the neuron differentiation (GO:0030182) and DNA repair (GO:0006281) pathway, and positively correlated with G4; one gene (ccl20b) is involved in the neutrophil chemotaxis (GO:0030593) pathway and is positively correlated with G2 (Supplementary Fig. 3d). It revealed that SFL improves insomnia caused by changes in neutrophil chemotaxis by activating DNA repair and neuromodulation pathways. These results demonstrate that SFL interferes with the expression of genes in these pathways, which is consistent with previous studies^35^. Hence, SFL may also exhibit important potential for promoting sleep. The correlation heat map obtained by calculating pearson correlation coefficient between key driving genes and metabolites. As shown in Fig. 5b, the correlation analysis revealed a positive association of seven genes (neurod1, msh2, msh3, recql4, ercc5, rad5lc, and rev3l) with a metabolite (acetylcholine chloride), and a negative association with three metabolites (linolenic acid, propionylcarnitine, and hyodeoxycholic acid). These findings underscore the close relationship between the changes in the insomnia-like phenotype and distinctly different metabolites as well as genes exhibiting differential expression. These observations demonstrate that changes in the insomnia-like phenotype are closely associated with significantly different metabolites and genes.

### RT-qPCR validation of key genes

**Fig. 6:**
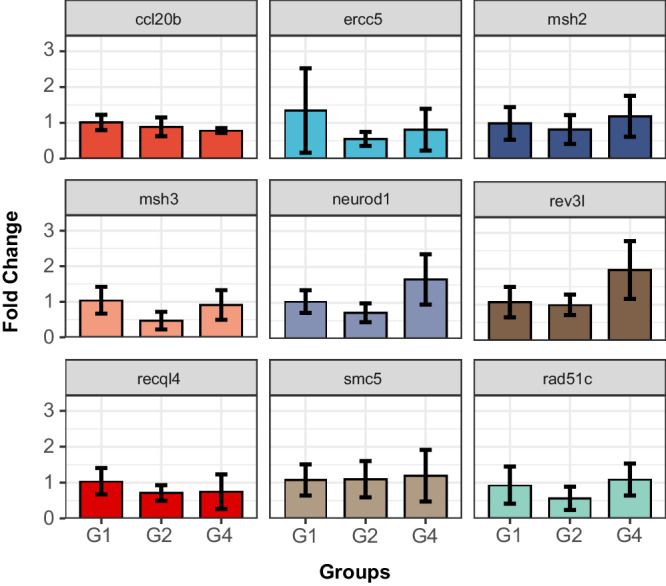
RT-qPCR validation of key genes in zebrafish; Data are presented as means ± standard deviation (s.d.) (*n* = 3).

To identify the results of the transcription profile, ten key driver genes were chosen for RT-qPCR verification. However, the polh gene was not expressed even after changing the primer sequence, which may be due to the low expression level. As depicted in Fig. 6, a total of seven genes demonstrated contrasting alterations in the G2 and G4 groups, and these genes were found to be associated with enriched pathways. The results demonstrated that the expression pattern of seven genes exhibited concurrence with that ascertained using RNA-seq, hence affirming the dependability of the RNA-seq data. The expression of neurod1, msh2, msh3, recql4, ercc5, rad5lc, smc5, and rev3l genes was downregulated in the G2 group and upregulated after G4, which may play a positive role in alleviating the insomnia symptoms. Combined with the GSEA-enriched pathway, it was observed that the regulation of SFL on insomnia-like phenotype in zebrafish was related to neuron differentiation and DNA repair pathway, respectively.

## Discussion

Sleep is essential for maintaining and restoring many physiological processes in the body^36,37^. Traditional Chinese medicine has unique advantages in the treatment of insomnia. The differences in locomotor behavior (moved distance and average speed) and neuroprotection between fermented GE-fed and model insomnia-like zebrafish demonstrated that fermented GE was able to effectively ameliorate PTZ-induced insomnia in zebrafish. Furthermore, significant changes in seven main active ingredients and the chemical profiling results based on LC–MS, as well as increased SCFA (significant changes in acetic acid and propanoic acid) suggest that changes in chemical composition was the driving force behind fermented GE-induced protection against PTZ-induced insomnia in zebrafish. Dietary fiber has been shown to be fermented by gut bacteria into short-chain fatty acids (acetate, propionate and butyrate). SCFAs may act as a key mediator in a series of neurological diseases through microbe-gut-brain communication^30^. In recent years, a number of studies have shown that SCFA (acetate, propionate and butyrate) can inhibit the release of inflammatory factors^38,39^. In addition, SCFA also affects the maturation and differentiation of central glial cells and regulates the development of central nervous inflammation^40^. Studies have also shown that sleep duration is positively correlated with acetate and propionate concentrations^30,41^. Our results show a significant increase in SCFA concentrations after fermentation, but detailed mechanistic studies are needed to elucidate the involvement and role of each SCFA in preventing insomnia in zebrafish. Evidence shows that the synergistic effect of probiotic fermentation of Chinese herbs can improve the effectiveness of Chinese herbs and contribute to the production of new bioactive substances during the fermentation process^42^, which may be the specific reason for the enhanced therapeutic effect of GE. However, the active ingredients of some Chinese herbs can only be absorbed after being transformed by gut microbes, and due to the complexity of the gut microbial network and the experimental limitations of this study, further microbial studies on fermented foods are needed to better understand the health benefits and potential mechanisms of fermented foods.

Comprehensive transcriptomic and metabolomic analysis showed that there were significant differences between the G1, G2, and G4 groups at the gene and metabolite levels. In the transcriptomics analysis, seven key genes associated with neuron differentiation and DNA repair pathways were identified. These seven genes are key genes in the neurodifferentiation and DNA repair pathway signaling pathway, indicating that SFL affects the expression of genes in this pathway. DNA damage repair increases during sleep and after external stimulation of DNA damage, and DNA damage in zebrafish neurons can trigger sleep, which may be a basic pathway related to insomnia^35^. Metabolomics analysis screened out four significantly different metabolites, including acetylcholine chloride, linolenic acid, propionylcarnitine, and hyodeoxycholic acid. Acetylcholine chloride and linolenic acid are closely related to sleep^43,44^. Four metabolites are primarily involved in the regulation of actin cytoskeleton, neuroactive ligand-receptor interaction, glycerophospholipid metabolism, alpha-linolenic acid metabolism, and biosynthesis of unsaturated fatty acids pathways. As mentioned earlier in section results, traditional Chinese medicine can improve insomnia through the top-ranked regulation of actin cytoskeleton pathway. These findings strongly suggest that these discovered metabolites and genes may be key regulators in the complex regulatory network of SFL on insomnia zebrafish. The above studies further confirm the preventive effect of SFL on insomnia, primarily attributed to its regulation of the actin cytoskeleton and DNA repair pathways. The findings suggest that the combined effect of significant metabolites and genes may contribute to the improvement of sleep disorders induced by PTZ-induced mental damage.

In conclusion, fermented GE effectively ameliorated PTZ-induced insomnia by reducing total distance and mean velocity of movement, improving neuronal apoptosis and regulating the transcription and metabolic pathways of multiple genes. Particularly, regulation of the actin cytoskeleton and DNA repair pathways were the crucial pathways of the anti-insomnia action of SFL. HPLC and LC-MS analysis showed that fermentation treatment significantly increased the content of five major types of compounds, including alkaloids, lipids, diterpenes, anthraquinones, and benzoyl derivatives, which might be responsible for the phenotypic differences. We then elucidated the pharmacological mechanism and potential therapeutic effect of SFL through the comprehensive application of transcriptomics and metabolomics. Our findings provide integrated omics information regarding the effect of fermented GE, a traditional Chinese Medicine ferment, on an insomnia model. Our results can be expected to be impactful, as the daily inclusion of fermented GE in a diet could be a dietary strategy to maintain sleep health. Our finding provides novel clues for treating insomnia by fermentation in GE. At present, the fermentation technology of Chinese herbal medicine is gradually mature, but the internal biotransformation mechanism of GE fermentation by probiotics is still unclear. This study provides the basis for the rational design of suitable fermentation strategies.

## Methods

### General

We purchased *Lactobacillus plantarum* from Yaxin Biotechnology in Taiwan. The saccharomyces cerevisiae (active dry yeast) was purchased from Angel Yeast Co.Ltd. Acetic acid bacteria (Hu Niang No. 1.01 Acetic acid bacteria) was purchased from Shanghai Difa Brewine-Biotechnology Co., Ltd. Gas chromatograph (Trace1300; Thermo Fisher Scientific, Waltham, MA, USA). LC-MS analysis was conducted using the 5600+ qTOF ESI MS system, which was manufactured by AB Sciex Pte. Ltd, located in MA, USA. Adenosine, gastrodin, 4-hydroxybenzyl alcohol, parishin A, B, C, and E were purchased from Shanghai Yuanye Biotechnology Co., Ltd. (Shanghai, China). Pentylenetetrazole (PTZ) was procured from Macklin Biochemical Technology Co. Ltd. (Shanghai, China). Oxazepam was bought from Yimin Pharmaceutical Factory (Beijing, China). AB wild adult zebrafish were procured from Shanghai FishBio Co. Ltd. (Shanghai, China). Energy Chemical Co. Ltd (Shanghai, China) supplied the organic solvents for the UPLC-QE-MS/MS.

### Sample preparation of GE at different processing stages

After fresh GE was cleaned, it was put into the fermentation tank, decocted with an appropriate amount of water for 90 min, and filtered to obtain GE extract (WGE). The specific experimental details of fermentation process refer to our previous research methods^45^. Next, the lactic acid bacteria strain solution was added to the sugar-sweetened WGE at the inoculation rate of 3% (V/V), after anaerobic fermentation at 37 °C for 24 h, and then the yeast strain solution was added. The prime fermentation liquid (PFL) was obtained after anaerobic co-fermentation at 28 °C for 15 days and sterilization at 60 °C for 30 min. Finally, an acetic acid bacteria strain solution was added to the PFL. After about 50 days of aerobic fermentation at 30 °C, the fermentation was completed and the secondary fermentation liquid was prepared. The supernatant of fermentation liquid at different processing stages was collected, and all water was removed by freeze-drying to obtain GE samples at different processing stages.

### Zebrafish locomotor behavior investigation

Zebrafish maintenance and embryo collection were carried out according to a reported protocol^46,47^. The Institutional Animal Care & Use Committee of Northwest A&F University approved the animal experimental protocol (Protocol Permit Number: XN2023-0902, 15 March 2022). The investigation of zebrafish mobility was conducted in accordance with the previously established procedure outlined by our research group^47^. The zebrafish larvae at 5 dpf were subjected to treatment with varying quantities (20 µg/ml and 80 µg/ml) of WGE, PFL, and SFL samples for 4 h. The zebrafish groups were individually allocated into separate compartments of a 6-well plate, followed by the addition of 2.5 mM PTZ. Following 10 min of adaptation, the larvae were observed and their behavior was recorded for durations of 30 and 50 min, respectively. The video was subjected to analysis using ethovision software, which was developed by Noldus Information Technology bv. in the Netherlands.

### Assessment of neuronal apoptosis

The method described in the literature was utilized to carry out our experiment^48^. In brief, zebrafish larvae at 5 dpf were subjected to treatment with varying quantities (20 and 80 µg/ml) of WGE, PFL, and SFL samples for 4 h. The zebrafish were segregated into distinct groups and thereafter introduced into individual compartments of a 6-well plate. Following this, a concentration of 2.5 mM PTZ was administered to each group for 10 min. Following that, the larvae were subjected to two rinses using phosphate-buffered saline (PBS) and subsequently exposed to a concentration of 5 mg/L AO in a light-restricted setting for 30 min. After undergoing three rounds of washing with PBS, the subjects were anesthetized with the administration of 0.016% tricaine in culture water. The Nikon SMZ25 fluorescent stereoscope, manufactured by Nikon Corporation in Tokyo, Japan, was used to observe apoptotic nerve cells.

### Quantitative analysis of the main medicinal components of fermented GE

The test method was slightly modified on the foundations of previous research^49^. Briefly, the fermentation broth (5 mL) was centrifuged in a sterile centrifuge tube at 5000 rpm for 10 min. The supernatant was collected and filtered through a 0.22 μm nylon filter, and 1 mL of the supernatant was analyzed by high-performance liquid chromatography (HPLC). Detection was performed using an Ultimate3000 HPLC-UV (Thermo Fisher Scientific, USA) with a Century SIL C18 column (250 × 4.6 mm, 5 μm). The mobile phase consisted of 0.1% phosphoric acid in water for phase A and acetonitrile for phase B. The injection volume was set at 20 μL, the flow rate was 1 mL/minute, the column temperature was set at 30 °C, and the detection wavelength was 220 nm for quantification of adenosine, 4-hydroxybenzyl alcohol, parishin B, parishin E, gastrodin, parishin A, and parishin C in WGE, PFL, and SFL. The elution gradient was as follows: 0–10.0 min, 3%–10% B; 10.0–15.0 min, 10%-12% B; 15.0–25.0 min, 12%-18% B; 25.0–40.0 min, 18% B; and 40–40.1 min, 18%–-3% B; 40.1–50.0 min, 3% B.

### Metabolite extraction and LC-MS analysis of GE at different processing stages

Three portions of WGE (WGE1, WGE2, WGE3) and PFL (PFL 1, PFL2, PFL3) 、SFL (SFL 1, SFL2, SFL3) freeze-dried samples were precisely weighed and were respectively dissolved in MeOH solution to prepare the sample solution with the concentration of 100 μg/mL. UPLC-ESI-MS analysis and metabolite extraction procedures were conducted using the protocols outlined in our previously published works^47^. Furthermore, the PLS-DA analysis was performed using the Metaboanalyst website (https://www.metaboanalyst.ca/, visited on July 2, 2023).

### Zebrafish Larvae preparation for transcriptome and metabolism analysis

In brief, the blank group (G1) was given E3 water, the model group (G2) was to add 2.5 mM PTZ based on G1, and the test group (G4) was given SFL (80 µg/ml) for 4 h, and then 2.5 mM PTZ was added for 10 min. On day five, zebrafish larvales were gathered. Before use, centrifuge tubes were prepared and weighed. Each tube held a single hundred fish, which were then given three rounds of clean water washing before being killed in liquid nitrogen. Lastly, the samples were kept for transcriptomic, metabolomic, and PCR analysis in a refrigerator at ‒80 °C.

### RNA sequencing and data analysis of Zebrafish larvae

Three separate biological replicates of the 5 dpf Zebrafish larvae of the G1, G2, and G4 groups were used. For details of the experiment, please refer to the previous paper of our study group^46^. ALL RNA-seq data analyses and gene set enrichment analysis (GSEA) were performed utilizing the Dr. TOM network platform (http://report.bgi.com), an internal, specially designed data mining system of the BGI. Enriched gene sets were allocated based on a nominal *p*-value of less than 0.05 and an FDR *q*-value of less than 0.25. The use of gene set enrichment analysis (GSEA) was used to uncover trends related to the overrepresentation of biological terms in the transcript.

### LC-MS/MS profiling of metabolism

For sample processing methods and condition parameter settings, please refer to the previously published articles by our team^46,50^. Next, we used the Oebiotech tools, a free online platform for data analysis (https://cloud.oebiotech.cn/task), to do pathway enrichment.

### Evaluation of short-chain fatty acids

The method for SCFA analysis was slightly modified according to previous reports^51^. The separation was performed using a Thermo TG-WAXMS capillary column with dimensions of 30 m in length, 0.25 mm in internal diameter, and a particle size of 5 μm. Gas chromatography equipment was employed for this purpose. The samples were subjected to centrifugation at a velocity of 15,000 revolutions per minute and a temperature of 4 °C for five minutes until a transparent supernatant became visible. Using a 0.45 μm pore size membrane, the supernatant from various fermentation stages was collected in a 1 mL syringe and filtered. The organic phase was gathered and subjected to the following gas chromatography analysis. The temperature was started at 100 °C and increased gradually over the next three minutes to 150 °C at 5 °C/minute, 220 °C at 70 °C/minute, and finally, to 220 °C, where it remained for three minutes. The carrier gas, helium, was employed at a flow rate of 2.0 mL/minute. The injection volume of the sample was 1 μL, and it was divided at a ratio of 1:10 at a temperature of 230 °C, with an inlet split flow rate of 20 ml/minute. The flame ion detector (FID) used for the GC had a temperature of 250 °C. To create the standard curve, the concentration was utilized as the abscissa and the peak area as the ordinate. The content was then computed using the standard curve.

### RT-qPCR analysis

RT-qPCR assays were utilized to validate the extent of gene expression. The three groups (G1, G2, and G4) were conducted consistently, following the sample preparation procedure outlined in the zebrafish Larvae preparation for transcriptome and metabolism analysis experiment. The RNA extraction process utilized AG RNAex Pro RNA kit, manufactured by Accurate Biotechnology (Hunan) Co., Ltd. The retrotranscription reactions were performed using the EVO M-MLV RT Mix Kit (Accurate Biotechnology Co., Ltd., China). The 5X gDNA Clean Reaction Mix reagent contained in the kit was employed to eliminate DNA, whereas the 5X Evo M-MLV RT Reaction Mix was utilized to perform the reverse transcription of RNA into DNA. The sequences of the primers, which were created using the Shenggong online primer creation tool, are provided in Supplementary Table 1. Lastly, the quantification of fluorescence was conducted utilizing the Premix Pro Taq HS qPCR kit, manufactured by Accurate Biotechnology Co., Ltd., located in China. The internal reference gene utilized was the β-actin gene from zebrafish. The 2^‒ΔΔCT^ method was utilized to evaluate the fold change of the genes that were being tested.

### Statistical analysis

Statistical analysis was performed using the program GraphPad Prism (version 8.0) developed by GraphPad program Inc. (La Jolla, CA, United States) and the R project (version 4.2.1). The data were presented in the form of mean values together with their corresponding standard deviations. A one-way analysis of variance (ANOVA) was employed to perform the analysis of variations among groups.

### Reporting summary

Further information on research design is available in the Nature Research Reporting Summary linked to this article.

### Supplementary information

Supplementary Material

Reporting Summary

## Data Availability

We declare that all data related to this study are included in this paper and its supplementary information.

## References

[1] Punnoose, A. R., Golub, R. M. & Burke, A. E. Insomnia. *JAMA* **307**, 2653 (2012). 10.1001/jama.2012.6219 22735439

[2] Zhou H, Lu S, Yu Z, Zhang J, Mei Z (2024). Mechanisms for the biological activity of Gastrodia elata Blume and its constituents: A comprehensive review on sedative-hypnotic, and antidepressant properties. Phytomedicine.

[3] Cappuccio FP, Miller MA (2017). Sleep and cardio-metabolic disease. Curr. Cardiol. Rep..

[4] Vaccaro A (2020). Sleep loss can cause death through accumulation of reactive oxygen species in the gut. Cell.

[5] Wang Z (2021). Gut microbiota modulates the inflammatory response and cognitive impairment induced by sleep deprivation. Mol. psychiatry.

[6] Brouwer A (2020). Impact of sleep deprivation and high-fat feeding on insulin sensitivity and beta cell function in dogs. Diabetologia.

[7] Nowak N (2021). Rapid and reversible control of human metabolism by individual sleep states. Cell Rep..

[8] Wang S, Zhao Y, Hu X (2023). Exploring the mechanism of Suanzaoren decoction in treatment of insomnia based on network pharmacology and molecular docking. Front. Pharmacol..

[9] Wang W (2022). Valerian essential oil for treating insomnia via the serotonergic synapse pathway. Front Nutr..

[10] Thomson F, Craighead M (2008). Innovative approaches for the treatment of depression: Targeting the HPA axis. Neurochem Res..

[11] Jaworek AK, Jaworek M, Szafraniec K, Wojas-Pelc A, Szepietowski J (2020). Melatonin and sleep disorders in patients with severe atopic dermatitis. Adv. Dermatol Allergol..

[12] Alboni S, Benatti C, Montanari C, Tascedda F, Brunello N (2013). Chronic antidepressant treatments resulted in altered expression of genes involved in inflammation in the rat hypothalamus. Eur. J. Pharmacol..

[13] Wang PS, Bohn RL, Glynn RJ, Mogun H, Avorn J (2001). Hazardous benzodiazepine regimens in the elderly: effects of half-life, dosage, and duration on risk of hip fracture. Am. J. Psychiatry.

[14] Lembke A, Papac J, Humphreys K (2018). Our other prescription drug problem. N. Engl. J. Med..

[15] Soong C., Burry L., Greco M. & Tannenbaum C. Advise non-pharmacological therapy as first line treatment for chronic insomnia. BMJ (Clinical *research**ed*.) **372**, n680 (2021). 10.1136/bmj.n680 33757960

[16] Liu Z (2016). Parishin C’s prevention of Aβ 1-42-induced inhibition of long-term potentiation is related to NMDA receptors. Acta Pharmaceutica Sin. B.

[17] Jou SB, Tsai CJ, Fang CY, Yi PL, Chang FC (2021). Effects of N 6 -(4-hydroxybenzyl) adenine riboside in stress-induced insomnia in rodents. J. sleep. Res..

[18] Jung JW (2006). Anxiolytic-like effects of *Gastrodia elata* and its phenolic constituents in mice. Biol. Pharm. Bull..

[19] Campo-Soria C, Chang Y, Weiss DS (2006). Mechanism of action of benzodiazepines on GABAA receptors. Br. J. Pharm..

[20] Zhang Y (2012). NHBA isolated from Gastrodia elata exerts sedative and hypnotic effects in sodium pentobarbital-treated mice. Pharmacol., Biochem., Behav..

[21] Zhu H-Y (2018). 4-Hydroxybenzyl alcohol derivatives and their sedative-hypnotic activities. RSC Adv..

[22] Zhang X (2023). Research advances in probiotic fermentation of Chinese herbal medicines. iMeta.

[23] Marco ML (2021). The International Scientific Association for Probiotics and Prebiotics (ISAPP) consensus statement on fermented foods. Nat. Rev. Gastroenterol. Hepatol..

[24] Long SM (2014). Identification of marine neuroactive molecules in behaviour-based screens in the larval zebrafish. Mar. drugs.

[25] Everson CA, Henchen CJ, Szabo A, Hogg N (2014). Cell injury and repair resulting from sleep loss and sleep recovery in laboratory rats. Sleep.

[26] Hu X (2023). Neuroprotective effect of melatonin on sleep disorders associated with Parkinsonas Disease. Antioxidants (Basel, Switzerland).

[27] Wang Z (2022). From function to metabolome: Metabolomic analysis reveals the effect of probiotic fermentation on the chemical compositions and biological activities of perilla frutescens leaves. Front. Nutr..

[28] Wu Z (2022). How steaming and drying processes affect the active compounds and antioxidant types of Gastrodia elata Bl. f. glauca S. chow. Food Res. Int. (Ott., Ont.).

[29] Jou S, Tsai C, Fang C, Yi P, Chang F (2021). Effects of N ^6^ ‐(4‐hydroxybenzyl) adenine riboside in stress‐induced insomnia in rodents. J. Sleep. Res..

[30] Magzal F (2021). Associations between fecal short-chain fatty acids and sleep continuity in older adults with insomnia symptoms. Sci. Rep..

[31] Xie L (2013). Sleep drives metabolite clearance from the adult brain. Sci. (N. Y., N. Y.).

[32] Wu R, Wang H, Lv X, Shen X, Ye G (2020). Rapid action of mechanism investigation of Yixin Ningshen tablet in treating depression by combinatorial use of systems biology and bioinformatics tools. J. Ethnopharmacol..

[33] da Silva JS, Dotti CG (2002). Breaking the neuronal sphere: regulation of the actin cytoskeleton in neuritogenesis. Nat. Rev. Neurosci..

[34] Subramanian A (2005). Gene set enrichment analysis: a knowledge-based approach for interpreting genome-wide expression profiles. Proc. Natl Acad. Sci. USA.

[35] Zada D (2021). Parp1 promotes sleep, which enhances DNA repair in neurons. Mol. Cell.

[36] Chaput J-P (2020). Sleep timing, sleep consistency, and health in adults: a systematic review. Appl. Physiol. Nutr. Metab..

[37] Matricciani L, Paquet C, Galland B, Short M, Olds T (2019). Children’s sleep and health: A meta-review. Sleep. Med. Rev..

[38] Vinolo MAR, Rodrigues HG, Nachbar RT, Curi R (2011). Regulation of Inflammation by Short Chain Fatty Acids. Nutrients.

[39] Freeman CR (2018). Impact of sugar on the body, brain, and behavior. FBL.

[40] Li J-M (2019). Dietary fructose-induced gut dysbiosis promotes mouse hippocampal neuroinflammation: a benefit of short-chain fatty acids. Microbiome.

[41] Shimizu Y (2023). Shorter sleep time relates to lower human defensin 5 secretion and compositional disturbance of the intestinal microbiota accompanied by decreased short-chain fatty acid production. Gut Microbes.

[42] Mukherjee, A., Breselge, S., Dimidi, E., Marco, M. L. & Cotter, P. D. Fermented foods and gastrointestinal health: underlying mechanisms. *Nat Rev Gastroenterol Hepatol* 1–19 10.1038/s41575-023-00869-x (2023). 10.1038/s41575-023-00869-x 38081933

[43] Niwa Y (2018). Muscarinic Acetylcholine Receptors Chrm1 and Chrm3 Are Essential for REM Sleep. Cell Rep..

[44] Liu Q, Shan Q (2022). Associations of α-linolenic acid dietary intake with very short sleep duration in adults. Front. Public Health.

[45] Zhu Y, Yuan Y, Wang J, Zhang C (2022). Antioxidant and cholate binding ability of Momordica charantia vinegar in vitro. China Brew..

[46] Zhang AN (2023). Metabolic regulation and antihyperglycemic properties of diet-derived PGG through transcriptomic and metabolomic profiling. Food Funct..

[47] Zhang XY (2022). Metabolomic navigated Citrus waste repurposing to restore amino acids disorder in neural lesion. Food Chem..

[48] Zhang S (2020). Anti-Parkinson’s disease activity of phenolic acids from Eucommia ulmoides Oliver leaf extracts and their autophagy activation mechanism. Food Funct..

[49] Gao Y (2022). Effects of processing technology in producing area on contents of six pharmacodynamic components of tianma (*Gastrodia elata* BI.). Chin. Arch. Traditional Chin. Med..

[50] Ren S (2023). Metabolic exploration of the developmental abnormalities and neurotoxicity of Esculentoside B, the main toxic factor in Phytolaccae radix. Food Chem. Toxicol..

[51] Lunken GR (2021). Prebiotic enriched exclusive enteral nutrition suppresses colitis via gut microbiome modulation and expansion of anti-inflammatory T cells in a mouse model of colitis. Cell Mol. Gastroenterol. Hepatol..

