# Supplementary material for: Sleep promoting and omics exploration on probiotics fermented Gastrodia elata Blume
Source: NPJ Sci Food. 2024 Jun 18;8:33. doi: 10.1038/s41538-024-00277-8 (PMC11189394; doi:10.1038/s41538-024-00277-8)
Supplement: Supplementary file 1 — Supplementary Material [file 41538_2024_277_MOESM1_ESM.pdf]

Sleep promoting and omics exploration on probiotics fermented *Gastrodia elata* Blume

Chao-Qi Zhang [a], Xu-Dong Zhang [a], Yan Wang [a], Yi-Han Liu [a], Cun-Li Zhang [a, b, *] and Qiang Zhang [a, b, *]

a Shaanxi Key Laboratory of Natural Products & Chemical Biology, College of Chemistry & Pharmacy, Northwest A&F University, Yangling 712100, China

b Key Laboratory of Edible Plant Enzyme R&D and Monitoring, Shaanxi Wuding Biotechnology Co., Ltd., Hanzhong 724400, China

*Correspondence: cunli_zhang@nwsuaf.edu.cn (C.Z.); zhangq@nwsuaf.edu.cn (Q.Z.)

Contents

Sleep promoting and omics exploration on probiotics fermented *Gastrodia elata* Blume 1

Abbreviation List 2

Supplementary Table 1 Primer Sequences for qRT-PCR 3

Supplementary Table 2 Profiles of the main chemical components in fermented GE. 4

Supplementary Table 3 Chemical components that changed significantly between groups before and after fermentation identified by LC-MS 5

Supplementar Table 4 Enrichment results of the key DMs induced by SFL 6

Supplementary Table 5 Significantly changed genes in related pathways 7

Supplementary Table 6 Key driving genes induced by SFL 8

Supplementary Figure 1 The number of chemical components significantly increased and decreased among the three groups of WGE, PFL, and SFL 9

Supplementary Figure 2 The amount of substances found in ESI positive (pos) and negative (neg) modes 10

Supplementary Figure 3 GSEA analysis based on the GO database 11

Supplementary Figure 4 GSEA analysis based on the GO database 13

Abbreviation List

DMs, differential metabolites

FC, Fold Change

KEGG, Kyoto Encyclopedia of Genes and Genomes

LC-MS/MS, Liquid Chromatography coupled to tandem Mass Spectrometry

CID, pubchem cid.

PCA, Principal Component Analysis

sPLS-DA, Sparse Partial Least Squares-Discriminant Analysis

GSEA, gene set enrichment analysis

SFL, Secondary fermentation liquid

PTZ, Pentylenetetrazole

G1, Zebrafish group fed with E3 water

G2, Zebrafish group fed with 2.5 mM PTZ

G4, Zebrafish group fed with 80 μg/mL SFL

Supplementary Table 1 Primer Sequences for qRT-PCR

**primer**	**5’ to 3’**	**length**
neurod1-F	CAAGTGTCACAGCAGCTCCCAAG	23
neurod1-R	TCTTCTTCCTCGTCGTCCTCATCC	24
msh2-F	AGAACGCCAGTAAGAGCAGCAAAG	24
msh2-R	CCAAGACGAACACCCACCACAC	22
rad51c-4F	AGTGATTGACAGCATCGCCTTCC	23
rad51c-R	CCGCCACTCTGTGTTGTGTAGC	22
ercc5-F	AGTTGGTGGTTCATTCTGCTGTGG	24
ercc5-R	TGCGAGTGTTGATGCTGGCTTC	22
ccl20b-F	CACAAAACTGGACGCAAGAACGAG	24
ccl20b-R	TGGTGAACATGCTCATCGTCTTCG	24
msh3-F	GGTTAGCACTCCTTCTCGCACTAC	24
msh3-R	AATGCCTCCTCCTCCATCTTACCC	24
recql4-F	AGGCTCTGGTGTCTCTGCTGAAG	23
recql4-R	CGGCTCGTCTCCTCTCGTCTG	21
rev3l-F	CTGCTGATGTTCTCAACCGTCTCG	24
rev3l-R	GCTTTCGCTTCTCTGCCGTCTC	22
smc5-F	TGTCCAGGTACAGGACACGTGGA	23
smc5-R	CTTAACACACAGGTTTGGTAATAT	24
polh-F	TGTGCGTCAGGCTGGAGGTC	20
polh-R	GTCAGGCTGTCGTTGGTCATCTTC	24
β-actin-F	CGTGCTGTCTTCCCATCCA	19
β-actin-R	TCACCAACGTAGCTGTCTTTCTG	23

Supplementary Table 2 Profiles of the main chemical components in fermented GE.

Component (μg/mL)	WGE	PFL	SFL
Adenosine	8.49±0.10c	11.15±0.09b	245.72±12.53a
Gastrodin	122.01±1.38b	113.97±0.54a	--
4-Hydroxybenzyl alcohol	21.74±0.13b	16.19±0.09b	429.36±13.8a
Parishin A	64.48±17.95a	36.99±0.27b	--
Parishin B	64.1±17.73b	42.48±0.31b	185.07±17.72a
Parishin C	50.82±13.96b	35.86±0.19b	147.24±6.18a
Parishin E	92.14±25.42b	61.15±0.44b	265.58±25.41a

Each value is expressed as the mean ± SD (n = 3). Means with different letters within a line are significantly different ($P < 0.05$).

Supplementary Table 3 Chemical components that changed significantly between groups before and after fermentation identified by LC-MS

Class		CID			
**superclass**	**direct_parent**	**PFL_vs_WGE_dn**	**PFL_vs_WGE_up**	**SFL_vs_WGE_dn**	**SFL_vs_WGE_up**
Benzenoids	Phenylacetaldehydes	998		998	
Benzenoids	Diphenylethers	5564			5564
Benzenoids	m-Xylenes	6896		6896	
Lipids and lipid-like molecules	Lineolic acids and derivatives	3931			3931
Lipids and lipid-like molecules	Very long-chain fatty acids	11197		11197	
Lipids and lipid-like molecules	Jasmonic acids	107126		107126	
Lipids and lipid-like molecules	Sesquiterpenoids	3989419		3989419	
Lipids and lipid-like molecules	Eudesmane, isoeudesmane or cycloeudesmane sesquiterpenoids	4655876		4655876	
Lipids and lipid-like molecules	Diterpenoids	10018535/10448477	9944781/14239484/ 45782962/57509505	10018535/ 10448477	9944781/45782962/ 57509505
Nucleosides, nucleotides, and analogues	Purine nucleosides	60961		60961	
Organic acids and derivatives	Sulfuric acid monoesters	8778	24761	8778	
Organic acids and derivatives	Alpha amino acids	9475		9475	247
Organic nitrogen compounds	Cholines	305		305	
Organic nitrogen compounds	Dialkylarylamines	949		949	
Organic oxygen compounds	O-glycosyl compounds	5988		5988	
Organic oxygen compounds	Alkyl-phenylketones	11092		11092	
Organic oxygen compounds	Polyethylene glycols	17472		17472	
Organic oxygen compounds	Phenolic glycosides	115067		115067	
Organoheterocyclic compounds	Nicotinamides	936		936	
Organoheterocyclic compounds	Indolines	10328		10328	
Organoheterocyclic compounds	Furanoquinolines	68085		68085	
Organoheterocyclic compounds	Oxepanes	4330530		4330530	
Phenylpropanoids and polyketides	3,4-dihydrocoumarins	660		660	
Phenylpropanoids and polyketides	7-hydroxycoumarins	5281426		5281426	
Phenylpropanoids and polyketides	Isoflavanols	51136422		51136422	
Benzenoids	Benzoic acid esters		3026		3026/8346
Benzenoids	Benzophenones		3102		3102
Benzenoids	Benzenesulfonic acids and derivatives		8485		
Benzenoids	Phenylphosphines and derivatives		13097		
Lipids and lipid-like molecules	Long-chain fatty acids		985		985/5281
Lipids and lipid-like molecules	Retinoids		5538		5538
Lipids and lipid-like molecules	Fatty amides		8214		8214
Organic nitrogen compounds	2-arylethylamines		774		774/3614
Organoheterocyclic compounds	Pyridinecarboxylic acids		938		
Organoheterocyclic compounds	Aminopyridines and derivatives		10439		10439
Organoheterocyclic compounds	Pyrazoles		15073		15073
Organoheterocyclic compounds	Thiochromenes		79633		79633
Organoheterocyclic compounds	2,2-dimethyl-1-benzopyrans		114703		
Phenylpropanoids and polyketides	8-prenylated isoflavanones		10001497		
Benzenoids	Benzamides				231459
Lipids and lipid-like molecules	Medium-chain fatty acids				2266
Organoheterocyclic compounds	Phthalic anhydrides				6811
Phenylpropanoids and polyketides	Coumarins and derivatives				11833
Phenylpropanoids and polyketides	Cinnamic acids				13245
Phenylpropanoids and polyketides	Stilbenes				185914

Supplementar Table 4 Enrichment results of the key DMs induced by SFL

Annotation	in set	set	in background	-lg(p-value)
Regulation of actin cytoskeleton	1	2	4	2.585249343
Neuroactive ligand-receptor interaction	1	2	40	1.58779944
alpha-Linolenic acid metabolism	1	2	44	1.546691026
Glycerophospholipid metabolism	1	2	52	1.474709459
Biosynthesis of unsaturated fatty acids	1	2	69	1.353075531
Regulation of actin cytoskeleton	1	2	4	2.585249343

Supplementary Table 5 Significantly changed genes in related pathways

#	NCBI ID	Name	#	NCBI	Name	#	NCBI	Name	#	NCBI	Name
1	100002946	cxcl8a	35	30194	alcama	69	404275	helt	103	563208	ccl38a.5
2	100003859	cxcl8b.3	36	30274	foxg1a	70	405856	wrnip1	104	565400	msh3
3	100004133	ddb2	37	30300	her2	71	406637	mre11a	105	565592	pds5b
4	100006951	nrg2a	38	30301	her4.2	72	406845	msh2	106	565714	wnt7aa
5	100148115	LOC100148115	39	30444	shhb	73	415151	rac2	107	566632	med12
6	100148329	LOC100148329	40	30478	ascl1b	74	415158	cycsb	108	566749	smc5
7	100148840	wnt7bb	41	30536	emx3	75	415182	cyp2aa4	109	567383	recql4
8	100149066	her4.4	42	30537	emx2	76	445296	babam1	110	567537	cxcl20
9	100149863	LOC100149863	43	30549	fgf3	77	447819	eya2	111	568191	wnt5a
10	100192217	ccl20a.3	44	30630	anos1a	78	447916	rrh	112	568326	ccl35.1
11	100330921	si:ch211-	45	322545	cyp2x10.2	79	449792	cryba1l2	113	569100	rev3l
12	100331800	zmp:0000000545	46	324185	fanci	80	450081	rad51c	114	571301	ungb
13	100332599	ascc3	47	325939	si:dkey-	81	494577	cox6c	115	571943	lhx4
14	100332722	wnt6a	48	326962	cox5ab	82	541336	zgc:112496	116	58118	bdnf
15	100334604	LOC100334604	49	326975	cox4i1	83	541371	en1b	117	58127	runx3
16	100534909	LOC100534909	50	334081	rnf168	84	541386	xpc	118	58146	hey2
17	100535363	cox7a2b	51	335889	emsy	85	541440	cyp2x6	119	60632	wnt3a
18	100535716	LOC100535716	52	336118	cox7c	86	541502	ercc5	120	619245	draxin
19	100536999	ccl38a.3	53	336550	rps3	87	550405	lhx9	121	678520	polh
20	100537421	cxcr5	54	338225	crygm4	88	550600	her13	122	678586	rtn4b
21	100538153	slx4	55	352930	gli1	89	553184	crybb3	123	678653	unc119b
22	101883994	LOC101883994	56	359832	tmtopsa	90	554101	cox5aa	124	751634	hes2.2
23	101887128	mbd4	57	359836	her15.1	91	554103	cox7a2a	125	790965	si:dkey-
24	108180207	LOC108180207	58	393578	ufl1	92	554142	rgrb	126	791563	wnt16
25	114411	neurog3	59	393599	ddb1	93	554361	lmx1ba	127	792198	her4.3
26	114415	neurod6b	60	393930	bhlhe22	94	555610	mcm9	128	794050	ccl38.1
27	114418	crybb1	61	394032	irx2a	95	556621	ccl34b.4	129	795788	ccl25b
28	114435	neurod2	62	402847	cyp2x7	96	558534	atrip	130	796252	cxl34b.11
29	140613	her9	63	402857	s1pr4	97	559147	hes2.1	131	797669	ccl39.6
30	140616	sox10	64	402914	her12	98	559656	acsl3b	132	81881	crx
31	140618	tfap2a	65	402986	cry-dash	99	559796	bhlhe23	133	83776	cry5
32	140621	casp3a	66	403019	irx1a	100	561155	si:ch73-			
33	30105	wnt5b	67	403064	atm	101	562283	prkdc			
34	30169	neurod1	68	403145	myd88	102	563152	ccl20b			

Supplementary Table 6 Key driving genes induced by SFL

#	NCBI ID	Name
1	100151570	LOC100151570
2	30169	neurod1
3	406845	msh2
4	450081	rad51c
5	541502	ercc5
6	563152	ccl20b
7	565400	msh3
8	566749	smc5
9	567383	recql4
10	569100	rev3l
11	678520	polh

Supplementary Figure 1 The number of chemical components significantly increased and decreased among the three groups of WGE, PFL, and SFL

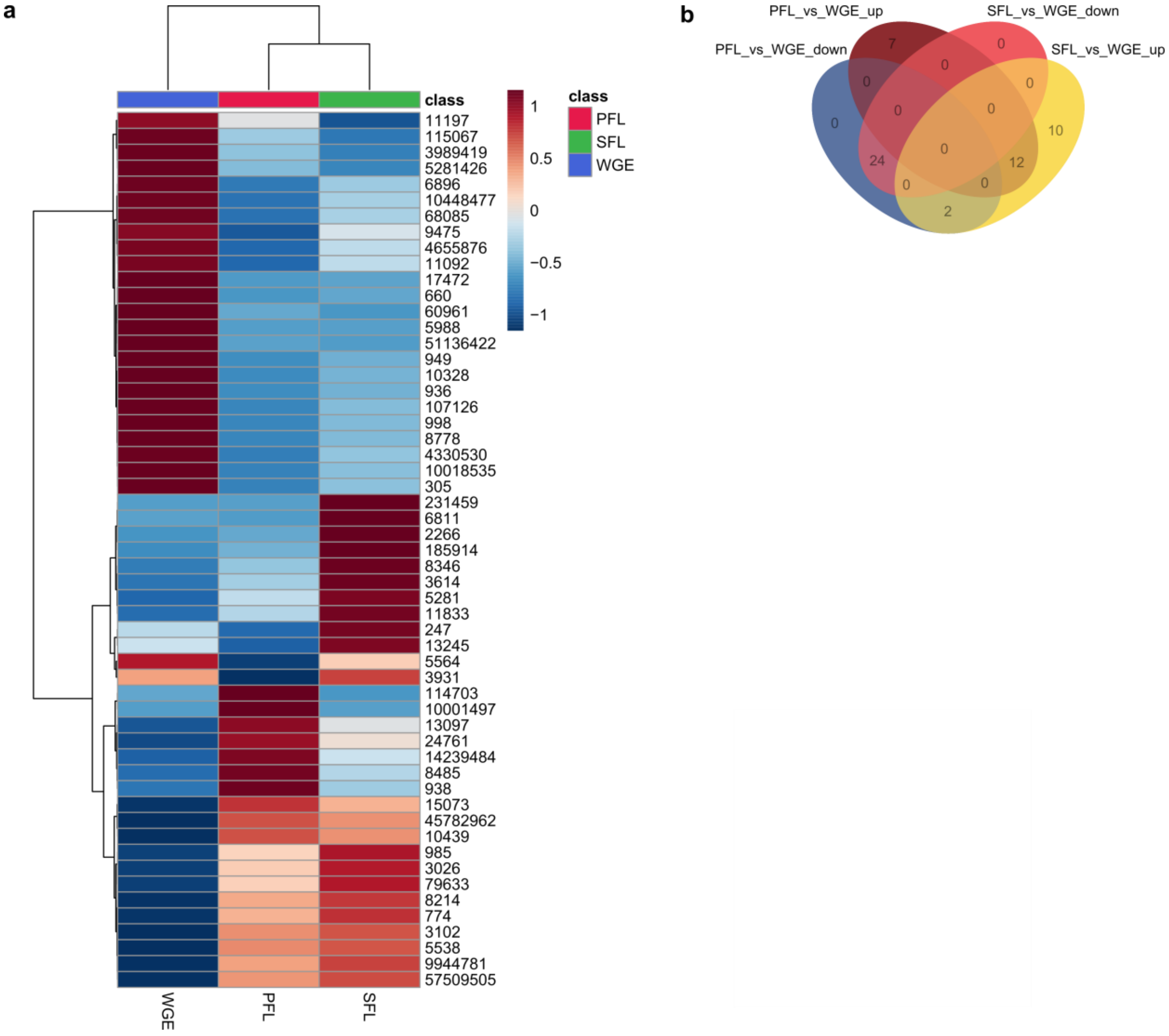

Supplementary Figure 1 The number of chemical components significantly increased and decreased among the three groups of WGE, PFL, and SFL. (**a**) Heat map of chemical composition content. The average value of three replicates of chemical components were used to make the heat map. (**b**) number of significantly changed chemical components are showed in a Venn diagram.

Supplementary Figure 2 The amount of substances found in ESI positive (pos) and negative (neg) modes

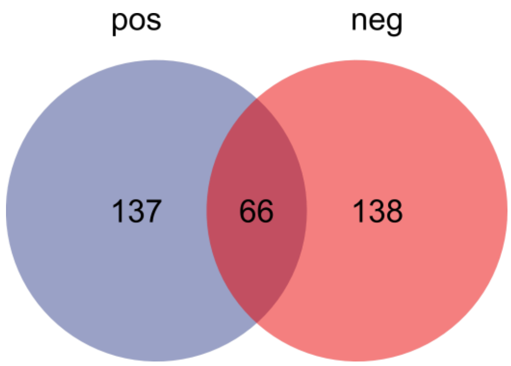

Supplementary Figure 3 GSEA analysis based on the GO database

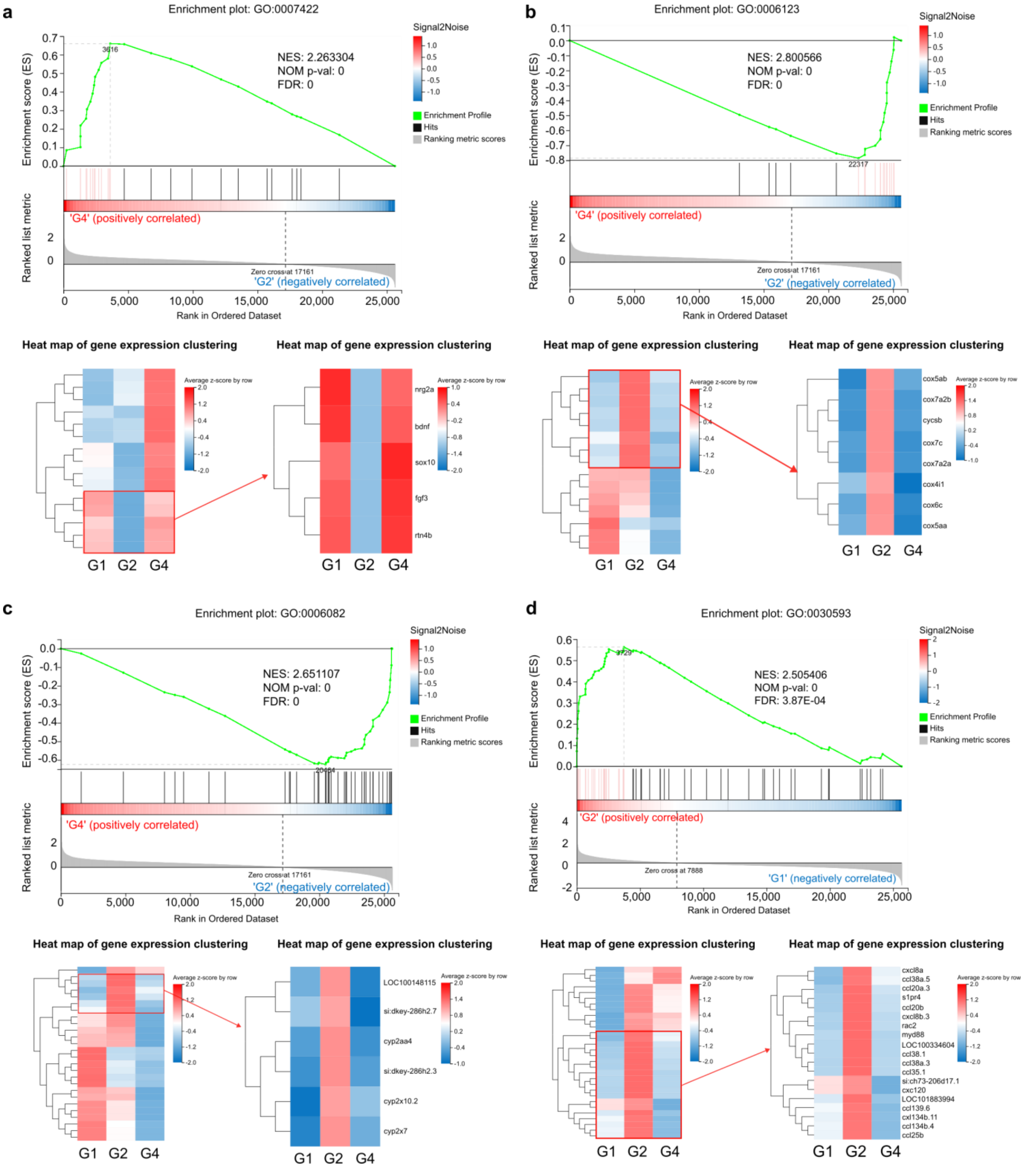

Results of GSEA based on the GO database. GSEA was performed in the G4 and G2 groups and G2 and G1 groups. In a ranked list of all the genes in the RNA-seq dataset, the GSEA algorithm generates an enrichment score that indicates the extent of overrepresentation at the top or bottom of the list of genes included in a gene set. Gene set enrichment at the top of the ranked list is indicated by a positive enrichment score (ES), whereas gene set enrichment at the bottom of the ranked list is indicated by a negative ES. Heatmaps beneath each enrichment plot show the expression heatmap signatures of genes involved in the GSEA analysis, referring to enrichment plots (**a**) to (**d**). Z scores were used. Left: the clustering of all genes within the pathway. Most sensitive pathways are highlighted at the right (indicated by a red square in panel 5). High expression is shown in red, and low expression is shown in green. The analysis demonstrates that known (**a**) peripheral nervous system development, while (**b**) Negative regulation of mitochondrial electron transport, cytochrome c to oxygen, and (**c**) Negative regulation of organic acid metabolic process are enriched in G2 groups, for the G4 vs G2 comparison group;(**d**) neutrophil chemotaxis is enriched in G2 groups.

Supplementary Figure 4 GSEA analysis based on the GO database

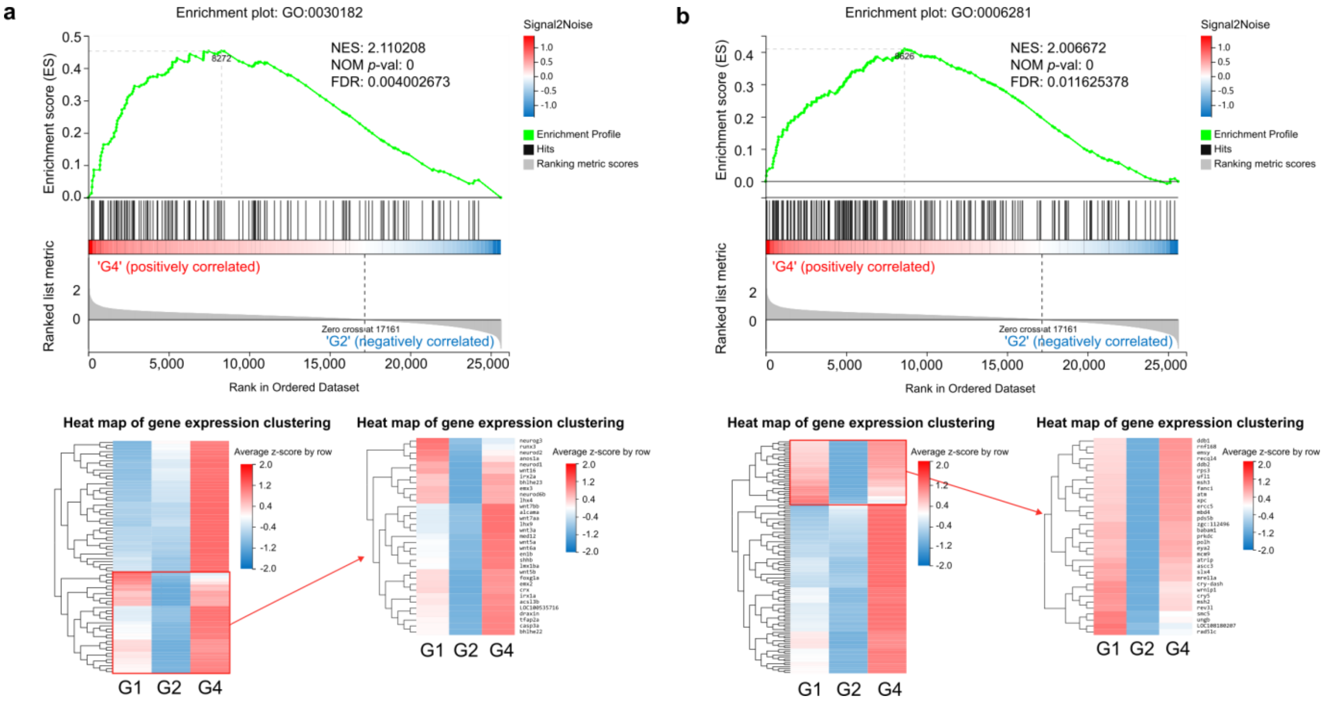

Results of GSEA based on the GO database. GSEA was performed in the G4 and G2 groups and G2 and G1 groups. In a ranked list of all the genes in the RNA-seq dataset, the GSEA algorithm generates an enrichment score that indicates the extent of overrepresentation at the top or bottom of the list of genes included in a gene set. Gene set enrichment at the top of the ranked list is indicated by a positive enrichment score (ES), whereas gene set enrichment at the bottom of the ranked list is indicated by a negative ES. Heatmaps beneath each enrichment plot show the expression heatmap signatures of genes involved in the GSEA analysis, referring to enrichment plots (**a**) to (**b**). Z scores were used. Left: the clustering of all genes within the pathway. Most sensitive pathways are highlighted at the right (indicated by a red square in panel 5). High expression is shown in red, and low expression is shown in green. The analysis demonstrates that known (**a**) regulation of neurogenesis, is enriched in G2 groups, while (**b**) Negative regulation of visual perception is enriched in G1 groups, for the G2 vs G1 comparison group.
